# Synergistic Effect of P Doping and Mo-Ni-Based Heterostructure Electrocatalyst for Overall Water Splitting

**DOI:** 10.3390/ma16093411

**Published:** 2023-04-27

**Authors:** Feihong Jia, Xiangyu Zou, Xueling Wei, Weiwei Bao, Taotao Ai, Wenhu Li, Yuchen Guo

**Affiliations:** School of Materials Science and Engineering, Shaanxi University of Technology, Hanzhong 723000, China

**Keywords:** P doping, nanoflower, heterostructure, synergistic effect, overall water splitting

## Abstract

Heterostructure construction and heteroatom doping are powerful strategies for enhancing the electrolytic efficiency of electrocatalysts for overall water splitting. Herein, we present a P-doped MoS_2_/Ni_3_S_2_ electrocatalyst on nickel foam (NF) prepared using a one-step hydrothermal method. The optimized P_[0.9mM]_-MoS_2_/Ni_3_S_2_@NF exhibits a cluster nanoflower-like morphology, which promotes the synergistic electrocatalytic effect of the heterostructures with abundant active centers, resulting in high catalytic activity for the hydrogen evolution reaction (HER) and oxygen evolution reaction (OER) in alkaline electrolyte. The electrode exhibits low overpotentials and Tafel slopes for the HER and OER. In addition, the catalyst electrode used in a two-electrode system for overall water splitting requires an ultralow voltage of 1.42 V at 10 mA·cm^−2^ and shows no obvious increase in current within 35 h, indicating excellent stability. Therefore, the combination of P doping and the heterostructure suggests a novel path to formulate high-performance electrocatalysts for overall water splitting.

## 1. Introduction

The excessive use of traditional fossil fuels has caused a series of problems, such as global warming and environmental pollution, and the dependence on nonrenewable energy sources has led to resource depletion [1,2]. Therefore, the exploitation of fresh and clean energy sources is an efficacious strategy to accomplish low-carbon emissions and mitigate environmental problems [3]. Hydrogen is an ideal clean fuel for solving energy and environmental issues [4,5]. Currently, most sustainable hydrogen processes are not clean enough; nevertheless, the electrochemical water-splitting method can be used more prevalently as a new approach to produce high-purity H_2_. However, the hydrogen evolution reaction (HER) and oxygen evolution reaction (OER) involve complex multi-electron transfer mechanisms, resulting in a thermodynamic potential of 1.23 V. The high potential is considered a major challenge for water splitting [6]. Noble metal Pt-based [7] and Ru/Ir-based oxides have been considered ideal HER and OER electrocatalysts because of their good catalytic intrinsic activity [8]. However, their high price, scarcity, and inferior stability limit their commercial applications [9,10]. Thus, the production of inexpensive water-splitting electrocatalysts with high catalytic activity, conductivity, and durability is highly desirable.

Currently, transition metal sulfides [11,12], (oxy)-hydroxides [13,14], nitrides [15,16], phosphides [15,17], and selenides [18], which enhance electrochemical performance and exhibit low overpotentials for water splitting, are preferable alternatives to noble metals. Among these electrocatalysts, nickel-sulfur compounds (NiS, NiS_2_, and Ni_3_S_2_) [19] have been used as OER catalysts because of their high conductivity, large specific surface areas, and Ni–Ni bond network structures of nickel sulfide (Ni_3_S_2_) [20]. However, poor HER activity and stability reduce their overall water-splitting efficiency [21,22]. Mo–S bonds are conducive to hydrogen chemisorption, and Mo-based sulfides are often used as typical HER catalysts [23,24]. The MoS_2_/Ni_3_S_2_ heterostructure can be engineered to reduce the overpotential and enhance the reaction kinetics [25,26]. For example, MoS_2_/Ni_3_S_2_-layered nanorods prepared by Yang et al. [25] formed a one-dimensional heterostructure of Mo-S-Ni, which promoted the overall water-splitting rate in electrocatalysis. However, MoS_2_/Ni_3_S_2_ still requires further modification to obtain electrocatalysts with high HER and OER activities.

Based on the construction of heterostructures, heteroatoms [27,28,29,30,31] are usually introduced to increase the number of active sites, optimize the electronic structure, and improve the reaction kinetics for the HER and OER. In recent years, V-doped Ni_3_S_2_ nanowire arrays (V-Ni_3_S_2_) have been prepared using a hydrothermal method [29], and the results showed that a large number of free carriers produced by V doping improved the catalytic performance of Ni_3_S_2_. Ma et al. [32] prepared N-doped hierarchical MoS_x_/Ni_3_S_2_-heterostructured nanowires on nickel foam (N-MoS_x_/Ni_3_S_2_-4@NF) for use as hydrogen evolution catalysts. They confirmed that N doping enhanced the conductivity and charge transfer rate of MoS_x_. P doping can expose more active sites and enhance the intrinsic conductivity and electrocatalytic activity of the catalysts [33,34]. Xue et al. [33] introduced P into the MoS_2_ matrix using the solid-phase ion exchange method, and the synthesized P-MoS_2_ catalyst exhibited good electrocatalytic performance for both the HER and OER. The P-MoS_2_/Mo_2_C electrocatalyst, which was prepared via high-temperature phosphating in a H_2_ atmosphere, exhibited highly efficient HER activity [34]. P doping also increases the number of active sites and reduces the adsorption free energy. Wang et al. [35] used P-induced electron density modulation to activate the basal plane of WS_2_ (P-WS_2_), and the HER performance of the P-WS_2_ nanowire arrays is superior to that of undoped WS_2_. Although the activity of these electrocatalysts has been vastly improved, P-doped electrocatalysts still require a lower overpotential for overall water splitting. Therefore, an effective approach to obtain highly efficient electrocatalysts is to further improve the synergistic effect of P doping and interface heterostructures, which could reduce the overpotential and increase the number of active sites for both the HER and OER processes.

Herein, a self-supporting P-doped MoS_2_/Ni_3_S_2_@NF heterostructure electrocatalyst was synthesized using a one-step hydrothermal method. Through the optimization and regulation of P doping, the synergistic effect of P doping and the interface heterostructure of the electrocatalyst resulted in remarkable electrocatalytic activity toward the overall water splitting. This study provides a new strategy for Mo-Ni-based electrocatalysts for efficient water electrolysis.

## 2. Experimental

### 2.1. Preparation of P_[xmM]_-MoS_2_/Ni_3_S_2_@NF and MoS_2_/Ni_3_S_2_@NF

(NH_4_)_2_MoS_4_ (41 mg) and (NH_4_)_2_HPO_4_ (0.3, 0.6, 0.9, 1.2, and 1.5 mM) were used as Mo, S, and P sources, respectively, which were fully dissolved in 35 mL DMF solvent by magnetic stirring and ultrasound to form a homogeneous solution. The solution and the pretreated carrier NF (1 × 4 cm^2^) were then transferred to a 50 mL polytetrafluoroethylene (Teflon)-lined stainless steel autoclave and heated at 200 °C for 12 h. Finally, the P_[xmM]_-MoS_2_/Ni_3_S_2_@NF catalyst electrodes with different initial P concentrations were obtained by repeatedly washing with deionized water and ethanol, and then drying at 60 °C for 10 h. A similar preparation method was used for MoS_2_/Ni_3_S_2_@NF, except that (NH_4_)_2_HPO_4_ was no longer added.

### 2.2. Preparation of MoS_2_@NF

When synthesizing MoS_2_ powder, the mixed solution of (NH_4_)_2_MoS_4_ (41 mg) and DMF (35 mL) was directly transferred to a Teflon-lined autoclave. After heating at 200 °C for 12 h, the black powder at the bottom of the autoclave was collected, washed several times, centrifuged, and dried. The MoS_2_ powder (7.13 mg) with the same loading as the target electrode was fully dissolved with ethanol, deionized water (V_H2O_/V_ethanol_ = 5/4), and Nafion binder (10 μL) to form a homogeneous solution, which was uniformly coated on NF (1 × 1 cm^2^) to prepare the MoS_2_@NF electrode.

### 2.3. Preparation of Ni_3_S_2_@NF

The preparation process is similar to that of P-MoS_2_/Ni_3_S_2_@NF. The NF was immersed in the homogeneous solution formed by CH_3_CSNH_2_ (91 mg) and DMF (35 mL), and then heated in a Teflon-lined stainless steel autoclave at 200 °C for 12 h. Finally, the Ni_3_S_2_@NF electrode was prepared after cleaning and drying.

## 3. Results and Discussion

### 3.1. Phase and Structural Analyses of P-MoS_2_/Ni_3_S_2_@NF

**Figure 1:**
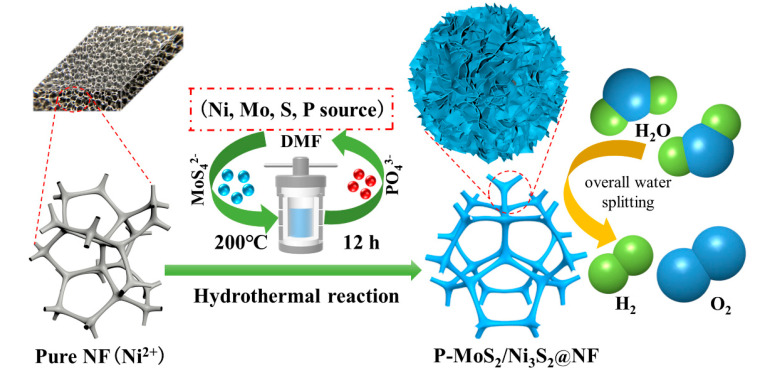
Schematic illustration of preparation process of P-MoS_2_/Ni_3_S_2_@NF electrocatalyst.

A simple one-step hydrothermal process was used to prepare the P-MoS_2_/Ni_3_S_2_@NF catalyst for the overall water splitting by controlling the initial concentration of the P doping. Figure 1 shows a schematic of the synthesis process. The three-dimensional porous NF structure (Figure S1g) displayed a role as a catalyst carrier and provided the Ni source, owing to its good electrical conductivity. The P-doped MoS_2_/Ni_3_S_2_@NF nanoflower heterostructure was formed via a hydrothermal reaction with MoS_4_^2−^ and PO_4_^3−^ in (NH_4_)_2_MoS_4_ as well as (NH_4_)_2_HPO_4_, respectively.

**Figure 2:**
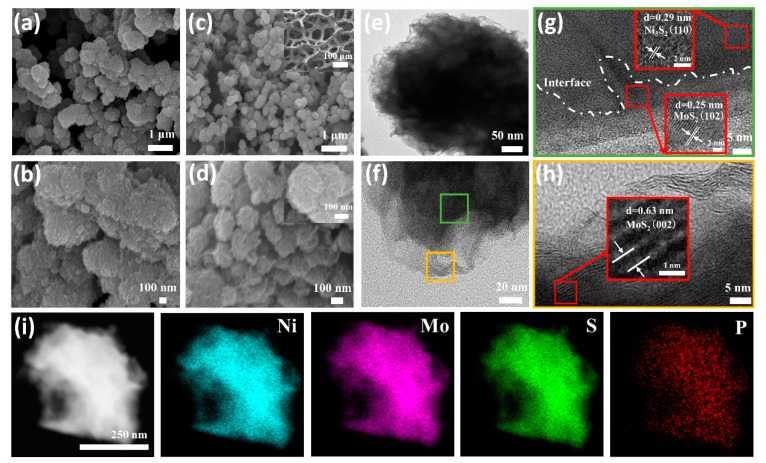
Low- and high-resolution SEM images: (**a**,**b**) MoS_2_/Ni_3_S_2_@NF, (**c**,**d**) P_[0.9mM]_-MoS_2_/Ni_3_S_2_@NF; insets show the NF skeleton and enlarged nanoflowers. (**e**,**f**) TEM image, (**g**,**h**) HRTEM image, with (**i**) STEM image and corresponding EDX element (Ni, Mo, S, P) mappings of P_[0.9mM]_-MoS_2_/Ni_3_S_2_@NF.

The surface morphology and structure of the catalysts were analyzed using scanning electron microscopy (SEM). The SEM image of the MoS_2_/Ni_3_S_2_@NF catalyst exhibits an irregular particle stacking structure (Figure 2a,b and Figure S1f). When the heteroatom P was introduced, the morphology and structure changed, and the low- and high-resolution SEM images of the P_[xmM]_-MoS_2_/Ni_3_S_2_@NF (x = 0.3, 0.6, 0.9, 1.2, and 1.5 mM) doped with different P concentrations are shown in Figure 2c,d and Figure S1a–e. When the initial P concentration was 0.3 mM, the granularity significantly decreased and lamellar growth was observed (Figure S1a). A small amount of nanoflowers (approximately 50 nm) began to form at a P concentration of 0.6 mM (Figure S1b). Finally, when the P concentration was increased to 0.9 mM, the nanoflowers were uniformly clustered to form a nanosphere heterostructure with a size of approximately 200 nm (Figure 2c,d and Figure S1c) and the spherical surface was distinctively covered with lamellae. As the P concentration increased, the nanoflowers gradually disappeared (Figure S1d) and finally became undulating cells (Figure S1e) owing to the excessive attachment. The results demonstrate that the content of P doping can affect the specific surface area of the catalytic materials [36], which can effectively modify the morphology and structure of Mo-Ni-based materials, while the high content of P doping changes the structure of the materials to a great extent [33]. In addition, the low-resolution SEM image in Figure S1g shows that the surface of the NF was initially smooth. However, the structure of the NF scaffold became rough after the P_[xmM]_-MoS_2_/Ni_3_S_2_ synthesis (Figure S1a–f) due to an increase in the number of active attachment sites on the catalyst. The internal structural characteristics of the P_[0.9mM]_-MoS_2_/Ni_3_S_2_@NF were further analyzed using transmission electron microscopy (TEM). Figure 2e,f and Figure S2a,b show typical nanoflower structures and the presence of irregular flakes around the sphere, which may be due to the low crystallinity of the crystal [34]. The crystal structures of the nanoflowers were further explored using high-resolution TEM (HRTEM). The lattice fringes of the inner and outer lamellar structures of the spheres are shown in Figure 2g,h and Figure S2c,d. The edge lattice fringes with the d-spacings of 0.25, 0.63, and 0.27 nm were calculated based on the figures, and the results correlate well with the corresponding d values of the (102), (002), and (100) crystal planes of the MoS_2_ phase (JCPDS No. 75-1539), respectively. The intermediate lattice fringes with the d-spacings of 0.29 nm are also consistent with the d value of the (110) crystal plane of the Ni_3_S_2_ phase (JCPDS No. 44-1418) [37]. Figure 2g shows the interface between the outer MoS_2_ and inner Ni_3_S_2_ crystals, which confirms that the nanoflower heterostructure consists of MoS_2_ nanosheets wrapped around Ni_3_S_2_. The Fast Fourier Transform (FFT) diffraction patterns (Figure S2e,f) of the P_[0.9mM]_-MoS_2_/Ni_3_S_2_@NF corresponding to the MoS_2_ and Ni_3_S_2_ single crystals were analyzed. In addition, the scanning TEM (STEM) image in Figure 2i and the corresponding energy-dispersive X-ray (EDX) spectroscopy elemental mapping confirmed the presence of Ni, Mo, S, and P in the P_[0.9mM]_-MoS_2_/Ni_3_S_2_@NF catalyst (Figure S3), which agrees with the TEM results.

**Figure 3:**
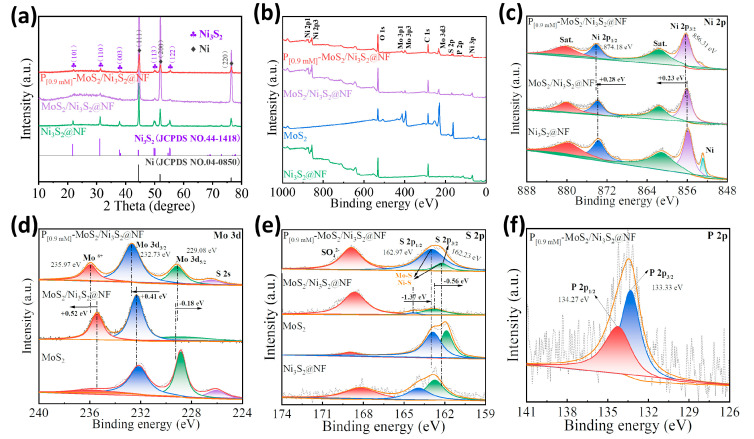
(**a**) XRD patterns of P_[0.9mM]_-MoS_2_/Ni_3_S_2_@NF, MoS_2_/Ni_3_S_2_@NF, and Ni_3_S_2_@NF electrodes. XPS of P_[0.9mM]_-MoS_2_/Ni_3_S_2_@NF (**b**) full spectrum, high-resolution spectra of (**c**) Ni 2p, (**d**) Mo 3d, (**e**) S 2p, and (**f**) P 2p.

The crystal-phase structure of the prepared P_[xmM]_-MoS_2_/Ni_3_S_2_@NF catalyst was characterized using X-ray diffraction (XRD), and the results are shown in Figure 3a and Figure S4a. The XRD patterns of the P_[0.9mM]_-MoS_2_/Ni_3_S_2_@NF, MoS_2_/Ni_3_S_2_@NF, and Ni_3_S_2_@NF catalyst electrodes are shown in Figure 3a. The characteristic peaks of the P_[0.9mM]_-MoS_2_/Ni_3_S_2_@NF at 2*θ* = 21.8°, 30.3°, 37.8°, 49.7°, and 55.2° are attributed to the (101), (110), (003), (113), and (122) crystal planes of the Ni_3_S_2_ phase (JCPDS No. 44-1418), respectively. Except for the remaining impurity peaks formed by molybdate oxidation, the phase diffraction peaks correlate well. The XRD spectrum of the carrier NF exhibits highly crystalline diffraction peaks at 44.6°, 52.0°, and 77.0° corresponding to the (111), (200), and (220) crystal planes of the Ni phase (JCPDS No. 04-0850), respectively. Figure S4b displays the XRD pattern of the MoS_2_ powder. Owing to the low crystalline state [30,38], the MoS_2_ diffraction peak is relatively broad, which is consistent with the HRTEM results. No obvious peak shift was observed between the Ni_3_S_2_ and Ni after P doping, indicating that the introduction of P did not change the crystal structure of the MoS_2_/Ni_3_S_2_.

The chemical composition and valence of the P_[0.9mM]_-MoS_2_/Ni_3_S_2_@NF catalyst were further analyzed by X-ray photoelectron spectroscopy (XPS). The full spectrum displays the presence of elements such as Ni, Mo, S, and P (Figure 3b), which is consistent with the EDX results. The high-resolution XPS spectrum of Ni 2p is shown in Figure 3c. The P_[0.9mM]_-MoS_2_/Ni_3_S_2_@NF exhibits strong Ni 2p_3/2_ and Ni 2p_1/2_ peaks at binding energies of 856.31 and 874.18 eV, respectively. The energy difference of 17.87 eV between the two peaks indicates the coexistence of the Ni^2+^ and Ni^3+^ oxidation states [39], in which “Sat” is the oscillating satellite peak formed by the Ni–O bond [40]. Compared with the single-component Ni_3_S_2_@NF, the Ni 2p_3/2_ and Ni 2p_1/2_ peaks of the MoS_2_/Ni_3_S_2_@NF and P_[0.9mM]_-MoS_2_/Ni_3_S_2_@NF show a positive shift. Compared with MoS_2_/Ni_3_S_2_@NF, the prominent Ni 2p_3/2_ and Ni 2p_1/2_ peaks continue to shift positively by 0.23 and 0.28 eV after P doping. It was confirmed that the MoS_2_/Ni_3_S_2_ heterostructure achieved interfacial charge transfer and distribution through the Mo–S–Ni bonds [41]. The high-resolution XPS spectrum of Mo 3d (Figure 3d) exhibits two main peaks of Mo^4+^ (Mo 3d_5/2_ and Mo 3d_3/2_) and Mo^6+^ [42,43]. The signals at 229.08, 232.73, and 235.97 eV correspond to the characteristic peaks of Mo 3d_5/2_, Mo 3d_3/2_, and Mo^6+^ of the P_[0.9mM]_-MoS_2_/Ni_3_S_2_@NF, respectively. Compared with the MoS_2_/Ni_3_S_2_@NF, the Mo 3d_3/2_ and Mo^6+^ peaks of the P_[0.9mM]_-MoS_2_/Ni_3_S_2_@NF show positive shifts of 0.41 and 0.52 eV, and the Mo 3d_5/2_ peaks show negative shifts of 0.18 eV. Furthermore, the intensity of the peak of Mo 3d_5/2_ is significantly enhanced, whereas that of Mo^6+^ is weakened, which may be attributed to the reduction in the Mo valence due to the formation of P–Mo bonds by P doping [44]. In addition, a small S 2s peak was observed at 226.42 eV owing to oxidation, indicating the formation of S–O bonds. Figure 3e displays the high-resolution S 2p spectrum. The binding energies of the characteristic S 2p_3/2_ and S 2p_1/2_ peaks of the P_[0.9mM]_-MoS_2_/Ni_3_S_2_@NF were 162.23 and 162.97 eV, respectively, indicating the formation of the S^2−^ corresponding metal sulfides at the Ni–S and Mo–S bond sites [45]. Compared to the single-component Ni_3_S_2_@NF and MoS_2_, the corresponding peaks of the target electrode exhibit a certain energy shift. In particular, the characteristic S 2p_3/2_ and S 2p_1/2_ peaks of the P_[0.9mM]_-MoS_2_/Ni_3_S_2_@NF negatively shifted by 0.56 and 1.37 eV, respectively, compared to the MoS_2_/Ni_3_S_2_@NF. In addition, the intensity of the S 2p_1/2_ peak is significantly enhanced, which corresponds to the results of the Mo 3d spectrum, indicating an increase in the Mo–S bond energy. In addition, a high-intensity peak of the residual SO_4_^2−^ species (168.88 eV) was formed owing to S oxidation. Compared with MoS_2_/Ni_3_S_2_@NF, the positive shift of the Ni 2p, Mo 3d_3/2_, and Mo^6+^ peaks and the negative shift of the Mo 3d_5/2_ and S 2p peaks indicate that there is charge transfer between the MoS_2_ and Ni_3_S_2_ interface, which is beneficial to improve the electrocatalytic reaction process [42]. Figure 3f exhibits the high-resolution P 2p spectrum. The P_[0.9mM]_-MoS_2_/Ni_3_S_2_@NF spectrum exhibits strong peak signals at 133.33 and 134.27 eV, which correspond to P 2p_3/2_ and P 2p_1/2_, respectively. This is possibly ascribed to the P–Mo and P–Ni bonds, which further verify the successful introduction of P into MoS_2_/Ni_3_S_2_@NF [33,46,47]. The XPS results show that there is a strong electronic interaction between the MoS_2_ and Ni_3_S_2_ species, suggesting that the establishment of the MoS_2_/Ni_3_S_2_ heterointerface and P doping are beneficial for further enhancing the electronic interaction and synergistically optimizing the electronic structure. In addition, P can regulate the charge redistribution at the interface of the MoS_2_/Ni_3_S_2_ heterostructure [34], thereby improving the charge transfer and reaction kinetics of Mo-S-Ni in electrocatalysis.

### 3.2. Electrocatalytic HER Activity

**Figure 4:**
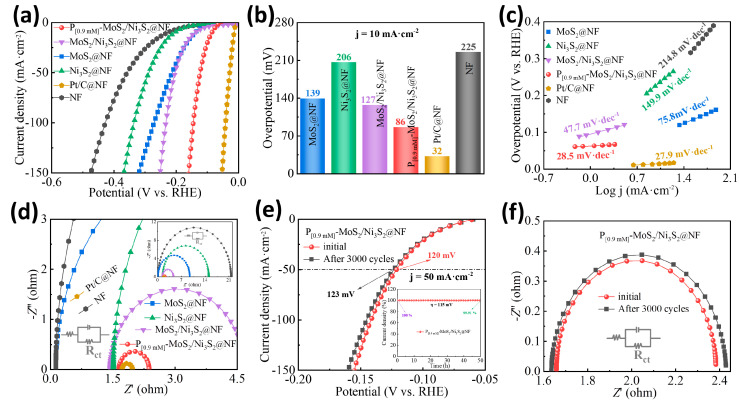
HER performance of different electrocatalysts: (**a**) LSV curve, (**b**) overpotential histogram at a current density of 10 mA·cm^−2^, (**c**) Tafel slope, and (**d**) Nyquist curve. Electrocatalytic HER stability of P_[0.9mM]_-MoS_2_/Ni_3_S_2_@NF: (**e**) LSV curves before and after 3000 cycles and chronoamperometry curves of insets, (**f**) Nyquist curves before and after 3000 cycles.

The HER performance of the P_[0.9mM]_-MoS_2_/Ni_3_S_2_@NF electrocatalysts was evaluated using a three-electrode system in 1 M KOH alkaline electrolyte, and the hydrogen evolution activities of MoS_2_@NF, Ni_3_S_2_@NF, MoS_2_/Ni_3_S_2_@NF, Pt/C@NF, and NF were tested for comparison. The obtained catalysts, a carbon rod, and Hg/HgO were used as the working, counter, and reference electrodes, respectively. Figure 4a displays the linear sweep voltammetry (LSV) curves of the various catalyst electrodes after iR correction. Other than the noble metal Pt/C@NF catalyst, which exhibited the best activity, the optimized P_[0.9mM]_-MoS_2_/Ni_3_S_2_@NF electrode also exhibited remarkable HER activity. When the same cathode current was reached, the applied driving voltage was the lowest and the current density increased sharply. Figure 4b exhibits the overpotential (*η*_10_) required for each electrode when the current density reached 10 mA·cm^−2^. The P_[0.9mM]_-MoS_2_/Ni_3_S_2_@NF exhibited a low overpotential of 86 mV, which is smaller than that of MoS_2_@NF (139 mV), Ni_3_S_2_@NF (206 mV), MoS_2_/Ni_3_S_2_@NF (127 mV), and NF (225 mV). The substantial improvement in the HER activity indicates that the heteroatom P doping promotes synergy between MoS_2_ and Ni_3_S_2_. The Tafel slopes of the electrodes were calculated and fitted (Figure 4c). Compared with MoS_2_@NF (75.8 mV·dec^−1^), Ni_3_S_2_@NF (149.9 mV·dec^−1^), MoS_2_/Ni_3_S_2_@NF (47.7 mV·dec^−1^), and NF (214.8 mV·dec^−1^), the P_[0.9mM]_-MoS_2_/Ni_3_S_2_@NF has the lowest Tafel slope of 28.5 mV·dec^−1^, which is close to the Tafel slope of the Pt/C@NF (27.9 mV·dec^−1^), and the lowest onset potential (*η_onset_*) of 61 mV. These results indicate that the kinetic activity of the HER is enhanced with an increase in the hydrogen production rate, which corresponds to the high-activity polarization curve shown in Figure 4a. The electrochemical impedance spectroscopy (EIS) curves in Figure 4d display the charge transfer resistance (R_ct_) of the different catalysts. It can be seen from the semicircle diameter of the fitted Nyquist curve that the R_ct_ of the P_[0.9mM]_-MoS_2_/Ni_3_S_2_@NF (0.73 Ω) is the smallest among the catalysts, except for Pt/C@NF. Thus, P doping is advantageous for accelerating electron transport on the NF surface for hydrogen evolution and improving the charge transfer kinetics in the HER, which correlates well with the electronic structure regulation measured by XPS [29,48]. The HER catalyst can be maintained with good stability, and Figure 4e displays the LSV curves of the P_[0.9mM]_-MoS_2_/Ni_3_S_2_@NF before and after 3000 cycles. When the driving current density reached 50 mA·cm^−2^, the *η*_50_ value of the polarization curve changed from 120 to 123 mV, the potential difference was small, and the curve was highly coincident. The electrode was also tested for long-term hydrogen evolution electrolysis at *η* = 115 mV for 50 h using chronoamperometry (Figure 4e). The stability curve of the electrolysis process exhibited a smooth trend, and the percentage change in the current density slightly decreased to 99.91%, indicating that the cathodic current loss was negligible. Based on the LSV results, the EIS curves of the P_[0.9mM]_-MoS_2_/Ni_3_S_2_@NF electrode before and after 3000 cycles were obtained. As shown in Figure 4f, the diameter of the fitted semicircle remained almost unchanged, with certain repeatability and coincidence. The smooth step obtained from the multi-current (Figure S7a) and multi-potential steps (Figure S7b) tests indicates that the catalyst has good stability and durability for the electrocatalytic HER and can meet the practical application requirements of the industry. The effects of different P-doping concentrations of the P_[xmM]_-MoS_2_/Ni_3_S_2_@NF catalysts on the HER performance were also explored. Figure S5 displays a comparison of the HER performance of the P_[0.3mM]_-MoS_2_/Ni_3_S_2_@NF, P_[0.6mM]_-MoS_2_/Ni_3_S_2_@NF, P_[0.9mM]_-MoS_2_/Ni_3_S_2_@NF, P_[1.2mM]_-MoS_2_/Ni_3_S_2_@NF, and P_[1.5mM]_-MoS_2_/Ni_3_S_2_@NF. After optimization, the P_[0.9mM]_-MoS_2_/Ni_3_S_2_@NF catalyst, with a P-doped amount of 0.9 mM, exhibited the best electrocatalytic HER activity. Based on these results, P doping can improve the activity of MoS_2_/Ni_3_S_2_@NF, and too little or too much P doping can slightly decrease the activity.

### 3.3. Electrocatalytic OER Activity

**Figure 5:**
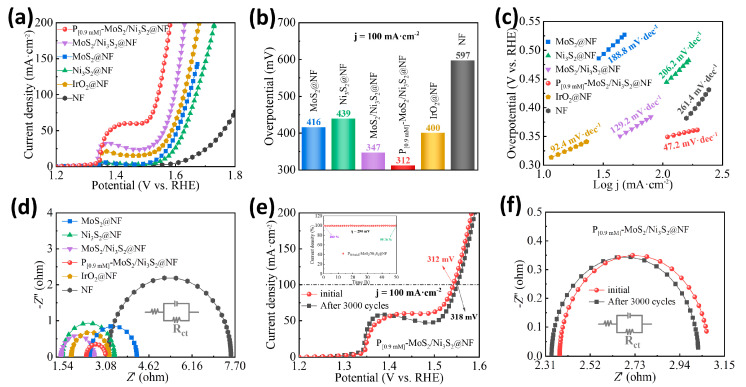
OER performance of different electrocatalysts: (**a**) LSV curve, (**b**) overpotential histogram at a current density of 100 mA·cm^−2^, (**c**) Tafel slope, and (**d**) Nyquist curve. Electrocatalytic OER stability of P_[0.9mM]_-MoS_2_/Ni_3_S_2_@NF: (**e**) LSV curves before and after 3000 cycles and chronoamperometry curves of insets, (**f**) Nyquist curves before and after 3000 cycles.

The OER performance of the P_[0.9mM]_-MoS_2_/Ni_3_S_2_@NF electrocatalyst was evaluated under identical conditions. Firstly, the P-doping concentration was optimized, and the electrocatalytic OER performances of the P_[0.3mM]_-MoS_2_/Ni_3_S_2_@NF, P_[0.6mM]_-MoS_2_/Ni_3_S_2_@NF, P_[0.9mM]_-MoS_2_/Ni_3_S_2_@NF, P_[1.2mM]_-MoS_2_/Ni_3_S_2_@NF, and P_[1.5mM]_-MoS_2_/Ni_3_S_2_@NF were evaluated (Figure S6). It was observed that the P_[0.9mM]_-MoS_2_/Ni_3_S_2_@NF exhibited good OER activity, which is consistent with the HER activity. The OER performances of the MoS_2_/Ni_3_S_2_@NF, MoS_2_@NF, Ni_3_S_2_@NF, IrO_2_@NF, and NF were further investigated, and the results are shown in Figure 5. Figure 5a displays the LSV curves of the catalyst electrodes. The P_[0.9mM]_-MoS_2_/Ni_3_S_2_@NF had the lowest *η_onset_*, and its anode current increased sharply with increasing driving voltage, even exceeding the performance of the noble metal IrO_2_@NF catalyst. Figure 5b displays the overpotential of each catalyst at a current density of 100 mA·cm^−2^ (*η*_100_). Compared with MoS_2_@NF (416 mV), Ni_3_S_2_@NF (439 mV), MoS_2_/Ni_3_S_2_@NF (347 mV), IrO_2_@NF (400 mV), and NF (597 mV), the P_[0.9mM]_-MoS_2_/Ni_3_S_2_@NF had the lowest overpotential of *η*_100_ = 312 mV. The Tafel slope of each catalyst is shown in Figure 5c. Compared with MoS_2_@NF (188.8 mV·dec^−1^), Ni_3_S_2_@NF (206.2 mV·dec^−1^), MoS_2_/Ni_3_S_2_@NF (129.2 mV·dec^−1^), and NF (261.4 mV·dec^−1^), the P_[0.9mM]_-MoS_2_/Ni_3_S_2_@NF had the smallest Tafel slope of 47.2 mV·dec^−1^. The EIS fitting curve in Figure 5d displays the R_ct_ values of the different electrodes. The semicircle diameter of the P_[0.9mM]_-MoS_2_/Ni_3_S_2_@NF was the smallest, affording a minimum impedance resistance of R_ct_ = 0.68 Ω. These results confirmed that P doping and MoS_2_/Ni_3_S_2_ heterostructures synergistically improve the charge transfer rate in the electrolyte [49]. The OER stability of the optimized P_[0.9mM]_-MoS_2_/Ni_3_S_2_@NF catalyst was also evaluated. The LSV (Figure 5e) and EIS (Figure 5f) curves before and after 3000 cycles were coincident, the *η*_100_ of the polarization curve increased from 312 to 318 mV at a current density of 100 mA·cm^−2^, and the potential difference was small. The oxygen evolution electrolysis test performed at *η* = 290 mV for 50 h using chronoamperometry is illustrated in Figure 5e. The stability curve of the electrolysis process exhibits a smooth and gradual downward trend; the percentage change in the current density decreases to 99.76%, whereas the anode current loss is negligible. The multi-current (Figure S7c) and multi-potential steps (Figure S7d) over a short period of time were also examined. It can be seen that the steps formed by the step current and potential are smooth and stable, indicating that the P_[0.9mM]_-MoS_2_/Ni_3_S_2_@NF has good stability and durability for the electrocatalytic OER. Table S1 summarizes and compares the HER and OER performances of the previously reported electrocatalysts. The performance of the P_[0.9mM]_-MoS_2_/Ni_3_S_2_@NF is better than that of most non-noble metal catalysts reported to date and it also exhibits good electrocatalytic activity for the HER and OER.

**Figure 6:**
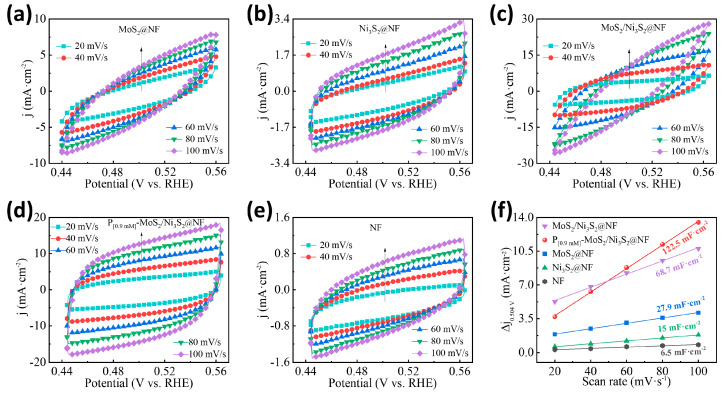
Comparison of different electrocatalysts ECSA curves of (**a**) MoS_2_@NF, (**b**) Ni_3_S_2_@NF, (**c**) MoS_2_/Ni_3_S_2_@NF, (**d**) P_[0.9mM]_-MoS_2_/Ni_3_S_2_@NF, (**e**) NF, and (**f**) C_dl_ curves.

To further explore the effect of P doping on the electrocatalytic active sites of the MoS_2_/Ni_3_S_2_@NF, the electrochemical active surface area (ECSA) test was conducted. The scanning speed of 20–100 mV/s and current density of $j_{0.504V}=\left(j_{a}-j_{b}\right)/2$ were used as the horizontal and vertical coordinates, respectively. The double-layer capacitance (C_dl_) values of the P_[xmM]_-MoS_2_/Ni_3_S_2_@NF, MoS_2_/Ni_3_S_2_@NF, MoS_2_@NF, Ni_3_S_2_@NF, and NF were obtained via calculations and fitting, and the results are shown in Figure S8 and Figure 6. Compared with the morphological and structural analyses by SEM and TEM, a large number of uniform flower-like spheres were formed on the surface of the P_[0.9mM]_-MoS_2_/Ni_3_S_2_@NF catalyst, which increased the number of exposed interfacial active sites and enlarged the active area. Therefore, the effective ECSA value obtained at the same scanning speed and potential range was larger, and the image was rounder and more symmetrical. The ECSA and C_dl_ curves of the P_[xmM]_-MoS_2_/Ni_3_S_2_@NF (Figure S8) demonstrate that as the P-doping concentration increases, the C_dl_ value of the P_[xmM]_-MoS_2_/Ni_3_S_2_@NF initially decreases and then increases to the maximum value of 122.5 mF·cm^−2^ (x = 0.9 mM), after which it decreases to 20.2 mF·cm^−2^ (x = 1.5 mM). The ECSA curves (Figure 6a–e) and C_dl_ values (Figure 6f) of the different catalysts show that the optimized P_[0.9mM]_-MoS_2_/Ni_3_S_2_@NF catalyst has the largest active surface area. Compared with the C_dl_ values of the MoS_2_/Ni_3_S_2_@NF (68.7 mF·cm^−2^), MoS_2_@NF (27.9 mF·cm^−2^), Ni_3_S_2_@NF (15.0 mF·cm^−2^), and NF (6.5 mF·cm^−2^), the P_[0.9mM]_-MoS_2_/Ni_3_S_2_@NF exhibits a maximum C_dl_ value of 122.5 mF·cm^−2^. The increase in the C_dl_ value indicates that the proper doping of heteroatom P can promote the uniform integration of MoS_2_ and Ni_3_S_2_, enriching a large number of active sites between the interfaces of the P_[0.9mM]_-MoS_2_/Ni_3_S_2_@NF catalyst, which increases the effective active area for electrocatalytic hydrogen production [50,51] and improves the electrocatalytic activity.

In summary, the P_[0.9mM]_-MoS_2_/Ni_3_S_2_@NF exhibited good electrocatalytic performance for the following reasons. The electronegativity of a P atom is less than that of a S atom, so P doping can produce a positive charge in a sulfide structure, and the lone pair electrons in a P atom can increase the electron spin density [52] and increase the active sites in the reaction process, thereby promoting the progress of the HER and OER. In addition, a TEM analysis shows that P doping can expose more edge structure defects, and the porous nanoflower structure forms a large specific surface area, which provides a sufficient reaction site for the HER and OER. It is worth noting that an SEM analysis shows that the introduction of a high concentration of P destroys the nanoflower structure of sulfides, thus reducing the electrical conductivity of Mo-Ni-based materials. Therefore, proper P content doping (2.06 wt%) can significantly improve the electrocatalytic performance of MoS_2_/Ni_3_S_2_. In addition, we also explored the electrocatalytic HER performance of the P_[0.9mM]_-MoS_2_/Ni_3_S_2_@NF in neutral (0.5 M PBS, pH = 7) and acidic (0.5 M H_2_SO_4_, pH = 0) electrolytes (Figure S9). Unfortunately, the performance of its electrocatalytic OER is poor, so further research is still needed.

### 3.4. Overall Water-Splitting Performance

**Figure 7:**
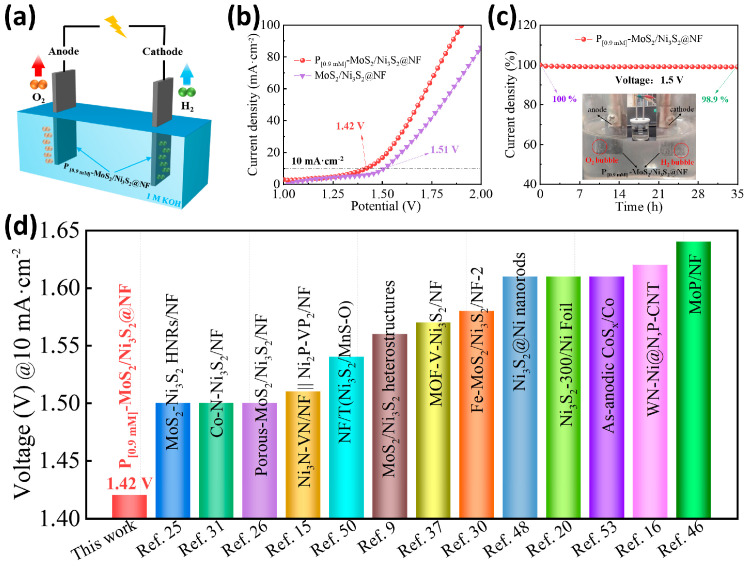
Overall water-splitting performance of P_[0.9mM]_-MoS_2_/Ni_3_S_2_@NF. (**a**) Schematic diagram of electrolyzed water, (**b**) polarization curve, (**c**) long-term durability curve measured by chronoamperometry, and (**d**) comparison with recently reported electrochemical overall water-splitting performance of bifunctional catalysts.

Based on the evaluation results of the electrocatalytic HER and OER (Figure S10 and Table S2), the P_[0.9mM]_-MoS_2_/Ni_3_S_2_@NF catalyst exhibits excellent activity for hydrogen and oxygen evolution. Thus, its performance as a bifunctional catalyst for overall water splitting in a two-electrode system of 1 M KOH alkaline electrolyte (Figure 7a) was examined. In the experiment, the P_[0.9mM]_-MoS_2_/Ni_3_S_2_@NF||P_[0.9mM]_-MoS_2_/Ni_3_S_2_@NF coupling electrode was used as the anode and cathode of the electrolysis cell. During the water-splitting process with an applied driving voltage, large amounts of H_2_ and O_2_ bubbles were continuously generated at the cathode and anode of the device diagram, respectively (Figure 7c). As shown in Figure 7b, the overall water-splitting polarization curve of the P_[0.9mM]_-MoS_2_/Ni_3_S_2_@NF reached 10 mA·cm^−2^ at a driving voltage of 1.42 V, and the combined overpotential of the electrocatalytic water-splitting process was only 190 mV, which is significantly lower than the voltage required for the MoS_2_/Ni_3_S_2_@NF electrolysis cell (1.51 V). The considerable decrease in the driving voltage indicates that the P_[0.9mM]_-MoS_2_/Ni_3_S_2_@NF catalyst promotes the reaction kinetics of the HER and OER [9]. The performance of the P_[0.9mM]_-MoS_2_/Ni_3_S_2_@NF catalyst is superior to that of most non-noble metal bifunctional catalysts previously reported (Figure 7d), including MoS_2_-Ni_3_S_2_ HNRs/NF (1.50 V) [25], Fe-MoS_2_/Ni_3_S_2_/NF-2 (1.61 V) [30], Ni_3_S_2_/MnS-O (1.54 V) [50], and as-anodic CoS_x_/Co (1.64 V) [53]. Figure 7c exhibits the chronoamperometry curve of the P_[0.9mM]_-MoS_2_/Ni_3_S_2_@NF operating for 35 h at 1.5 V. The results indicate that the electrolysis cell provides a stable current. During the long-term water-splitting process, the percentage change in the cathode and anode current densities was 98.9%, indicating that the water-splitting performance decreased slightly by 1.1%. The morphology of the P_[0.9mM]_-MoS_2_/Ni_3_S_2_@NF after overall water splitting was also examined using SEM (Figure S11a,b), showing that most of the nanoflower structures were retained. The EDX elemental mapping spectrum (Figure S11c–g) confirmed the uniform distribution of Ni, Mo, S, and P on the catalyst surface. In conclusion, the P_[0.9mM]_-MoS_2_/Ni_3_S_2_@NF exhibits excellent overall water-splitting performance and stability in alkaline solution.

**Figure 8:**
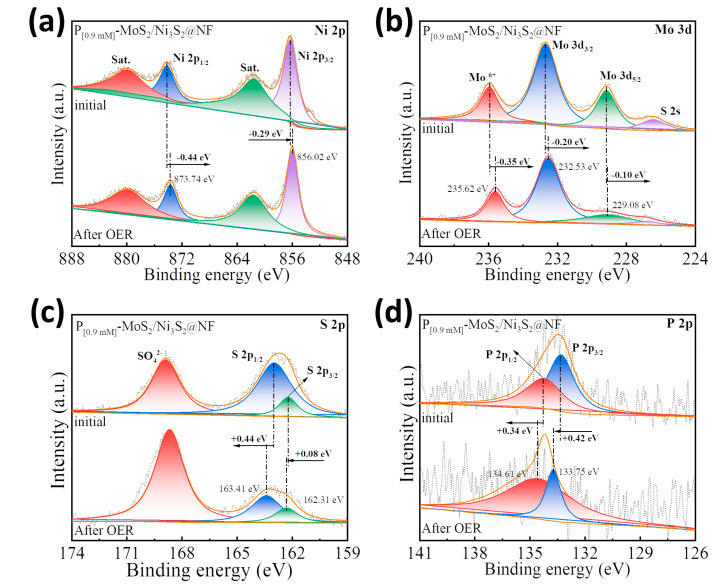
XPS comparison before and after electrochemical OER testing of P_[0.9mM]_-MoS_2_/Ni_3_S_2_@NF: high-resolution spectra of (**a**) Ni 2p, (**b**) Mo 3d, (**c**) S 2p, and (**d**) P 2p.

In order to explore the mechanism of P doping in the electrocatalytic reaction process, the P_[0.9mM]_-MoS_2_/Ni_3_S_2_@NF was characterized by XPS after a long-term OER test (Figure 8). The XPS full spectrum after the test correlates well with that before the test (Figure S12). Compared to the high-resolution spectrum of the initial P_[0.9mM]_-MoS_2_/Ni_3_S_2_@NF sample, the corresponding characteristic peaks of the Ni 2p (Figure 8a) and Mo 3d (Figure 8b) spectra shifted to the low-energy direction and became electron-rich centers. In contrast, the corresponding characteristic peaks of the S 2p (Figure 8c) and P 2p (Figure 8d) spectra shifted to the high-energy direction and became electron-poor centers. In Figure 8c, the intensity of the S 2p_3/2_ and S 2p_1/2_ peaks was significantly weakened, which may be attributed to the leaching of S during the electrocatalytic process. Compared with the spectrum before the test, the characteristic P 2p_3/2_ (133.75 eV) and P 2p_1/2_ (134.61 eV) peaks of the P_[0.9mM]_-MoS_2_/Ni_3_S_2_@NF after testing were positively shifted by 0.42 and 0.34 eV, respectively (Figure 8d). The above results show that the S atom with weaker electronegativity has a charge exchange with Ni and Mo with stronger electronegativity, which optimizes the internal electronic structure of MoS_2_ and Ni_3_S_2_. At the same time, the P atom with the weakest electronegativity also has electron transfer, and the electrons transfer from the P atom to Ni, Mo, and S, so that P has more positive charge, resulting in an increase in the OER active center material [54]. It is confirmed that P doping is beneficial to promote the synergistic effect of MoS_2_ and Ni_3_S_2_ in the OER process. In summary, the optimized electronic configuration due to P doping contributed to the enhanced electrocatalytic performance of the P_[0.9mM]_-MoS_2_/Ni_3_S_2_@NF heterostructure.

## 4. Conclusions

In summary, a P-doped MoS_2_/Ni_3_S_2_@NF heterostructure electrocatalyst was synthesized using a facile one-step hydrothermal method. The results of various tests and characterizations showed that the nanoflowers morphology formed by the P_[0.9mM]_-MoS_2_/Ni_3_S_2_@NF exposed more active sites, resulting in a large specific surface area and abundant active centers. In addition, the introduction of P promoted the synergistic effect of the MoS_2_/Ni_3_S_2_ heterostructure interface, optimized the electronic structure, and effectively improved the transport speed of the carriers in alkaline media. The combination of these engineering system properties synergistically enhanced the electrocatalytic performance and reaction kinetics of the P_[0.9mM]_-MoS_2_/Ni_3_S_2_@NF catalyst for the HER, OER, and overall water splitting. When used as an electrode in an electrolysis cell, P_[0.9mM]_-MoS_2_/Ni_3_S_2_@NF only requires an ultralow driving voltage of 1.42 V for water splitting at 10 mA·cm^−2^, which is superior to that of most bifunctional electrocatalysts reported to date. This study opens a novel avenue for designing highly active, stable, and economical multi-component P-doped heterostructure electrocatalysts.

## Data Availability

Data sharing is not applicable to this article as no new data were created or analyzed in this study.

## Supplementary Materials

The following supporting information can be downloaded at https://www.mdpi.com/article/10.3390/ma16093411/s1, Figure S1: Low- and high-resolution SEM images: (a) P_[0.3mM]_-MoS_2_/Ni_3_S_2_@NF, (b) P_[0.6mM]_-MoS_2_/Ni_3_S_2_@NF, (c) P_[0.9mM]_-MoS_2_/Ni_3_S_2_@NF, (d) P_[1.2mM]_-MoS_2_/Ni_3_S_2_@NF, (e) P_[1.5mM]_-MoS_2_/Ni_3_S_2_@NF, (f) MoS_2_/Ni_3_S_2_@NF, and (g) carrier NF; Figure S2: (a,b) TEM images, (c,d) HRTEM images, and corresponding (e,f) FFT patterns of P_[0.9mM]_-MoS_2_/Ni_3_S_2_@NF; Figure S3: The EDX spectrum of P_[0.9mM]_-MoS_2_/Ni_3_S_2_@NF and the corresponding atomic (Ni, Mo, S, P) contents; Figure S4: The XRD patterns of (a) catalyst electrodes doped with different P concentrations and (b) MoS_2_ powders; Figure S5: Comparison of HER performance of catalyst electrodes doped with different P concentrations: (a) LSV curve, (b) overpotential histogram at a current density of 10 mA·cm^−2^, (c) Tafel slope, and (d) Nyquist curve; Figure S6: Comparison of OER performance of catalyst electrodes doped with different P concentrations: (a) LSV curve, (b) overpotential histogram at a current density of 100 mA·cm^−2^, (c) Tafel slope, and (d) Nyquist curve; Figure S7: Multi-current and multi-potential steps for (a,b) HER, (c,d) OER; Figure S8: Comparison of electrocatalysts with different P-doping concentrations, ECSA curves of (a) P_[0.3mM]_-MoS_2_/Ni_3_S_2_@NF, (b) P_[0.6mM]_-MoS_2_/Ni_3_S_2_@NF, (c) P_[0.9mM]_-MoS_2_/Ni_3_S_2_@NF, (d) P_[1.2mM]_-MoS_2_/Ni_3_S_2_@NF, (e) P_[1.5mM]_-MoS_2_/Ni_3_S_2_@NF, and (f) C_dl_ curves; Figure S9: The electrocatalytic HER performance LSV curves of P_[0.9mM]_-MoS_2_/Ni_3_S_2_@NF in (a) neutral (0.5 M PBS, pH = 7) and (b) acidic (0.5 M H_2_SO_4_, pH = 0) electrolytes; Figure S10: Comparison of overpotentials and Tafel slopes of as-prepared catalysts doped with different P concentrations and single-component (a) HER, (b) OER; Figure S11: After electrochemical testing of P_[0.9mM]_-MoS_2_/Ni_3_S_2_@NF: (a,b) SEM images, (c–g) EDX mapping spectrum of corresponding elements (Ni, Mo, S, P); Figure S12: XPS full spectrum comparison before and after electrochemical OER testing of P_[0.9mM]_-MoS_2_/Ni_3_S_2_@NF; Table S1: Comparison of the electrocatalytic HER and OER performance of P_[0.9mM]_-MoS_2_/Ni_3_S_2_@NF and recently reported catalysts in alkaline electrolyte; Table S2: Comparison of R_ct_ and C_dl_ of prepared catalysts with different P concentrations and single-component catalysts. Refs [9,15,20,25,26,29,30,31,32,37,42,49,50,51,53,55] are cited in the supplementary materials.
