# Supplementary material for: Synergistic Effect of P Doping and Mo-Ni-Based Heterostructure Electrocatalyst for Overall Water Splitting
Source: Materials (Basel). 2023 Apr 27;16(9):3411. doi: 10.3390/ma16093411 (PMC10179828; doi:10.3390/ma16093411)
Supplement: Supplementary file 1 [file materials-16-03411-s001.zip › materials-2321032-supplementary.pdf]

List of Contents

1. **Experimental Section:**

1.1 Materials

1.2 Preparation of Pt/C@NF and IrO_2@NF

1.3 Material characterization

1.4 Electrochemical measurements

2. **Supplementary Figures:**

Figure S1. Low and high-resolution SEM images (a) $P_{[0.3\ mM]}$-MoS_2/Ni_3S_2@NF, (b) $P_{[0.6\ mM]}$-MoS_2/Ni_3S_2@NF, (c) $P_{[0.9\ mM]}$-MoS_2/Ni_3S_2@NF, (d) $P_{[1.2\ mM]}$-MoS_2/Ni_3S_2@NF, (e) $P_{[1.5\ mM]}$-MoS_2/Ni_3S_2@NF, (f) MoS_2/Ni_3S_2@NF and (g) carrier NF.

Figure S2. (a-b) TEM images, (c-d) HRTEM images, and corresponding (e-f) FFT patterns of $P_{[0.9\ mM]}$-MoS_2/Ni_3S_2@NF.

Figure S3. The EDX spectrum of $P_{[0.9\ mM]}$-MoS_2/Ni_3S_2@NF and the corresponding atomic (Ni, Mo, S, P) contents.

Figure S4. The XRD patterns of (a) catalyst electrodes doped with different P concentrations and (b) MoS_2 powders.

Figure S5. Comparison of HER performance of catalyst electrodes doped with different P concentrations (a) LSV curve, (b) overpotential histogram at a current density of 10 $mA\cdot cm^{-2}$, (c) Tafel slope, and (d) Nyquist curve.

Figure S6. Comparison of OER performance of catalyst electrodes doped with different P concentrations (a) LSV curve, (b) overpotential histogram at a current density of 100 $mA\cdot cm^{-2}$, (c) Tafel slope, and (d) Nyquist curve.

Figure S7. Multi-current and multi-potential steps (a-b) HER, (c-d) OER.

Figure S8. Comparison of electrocatalysts with different P doping concentrations, ECSA curves of (a) $P_{[0.3\ mM]}$-MoS_2/Ni_3S_2@NF, (b) $P_{[0.6\ mM]}$-MoS_2/Ni_3S_2@NF, (c) $P_{[0.9\ mM]}$-MoS_2/Ni_3S_2@NF, (d) $P_{[1.2\ mM]}$-MoS_2/Ni_3S_2@NF, (e) $P_{[1.5\ mM]}$-MoS_2/Ni_3S_2@NF, and (f) C_{dl} curves.

Figure S9. The electrocatalytic HER performance LSV curves of $P_{[0.9\ mM]}$-MoS_2/Ni_3S_2@NF in (a) neutral (0.5 M PBS, pH=7) and (b) acidic (0.5 M H_2SO_4, pH =0) electrolytes.

Figure S10. Comparison of overpotentials and Tafel slopes of as-prepared catalysts doped with different P concentrations and single-component (a) HER, (b) OER.

Figure S11. After electrochemical testing of $P_{[0.9\ mM]}$-MoS_2/Ni_3S_2@NF (a-b) SEM images, (c-g) EDX mapping spectrum of corresponding elements (Ni, Mo, S, P).

Figure S12. XPS full spectrum comparison before and after electrochemical OER testing of $P_{[0.9\ mM]}$-MoS_2/Ni_3S_2@NF.

3. Supplementary Tables:

Supplementary Table S1 Comparison of the electrocatalytic HER and OER performance of $P_{[0.9\ mM]}$-MoS_2/Ni_3S_2@NF and recently reported catalysts in alkaline electrolyte.

Supplementary Table S2 Comparison of R_{ct} and C_{dl} of prepared catalysts with different P concentrations and single-component catalysts.

4. Notes and references

1. Experimental section

1.1 Materials

Ammonium tetrathiomolybdate ($(NH_4)_2MoS_4$, 99.95% ATTM), diammonium hydrogen phosphate ($(NH_4)_2HPO_4$) and thioacetamide (CH_3CSNH_2) were purchased from Shanghai Aladdin Biochemical Technology Co. Ltd. N,N-dimethylformamide (DMF), hydrochloric acid (HCl) and potassium hydroxide (KOH) were purchased from Shanghai Macklin Biochemical Technology Co. Ltd. Perfluorosulfonic acid (Nafion) adhesive was purchased from Tianjin Aiweixin Chemical Technology Co. Ltd. Commercial IrO_2 and Pt/C were purchased from Shanghai Saen Chemical Technology Co. Ltd. Nickel foam (NF, wire diameter 1 mm, bulk density 0.45 g/cm^3, porosity 96%) was purchased from Kunshan Guangjiayuan New Materials Co. Ltd. All reagents were used at the time of reception without further purification. The deionized water used in the solution preparation and washing steps comes from ultrapure water (Milli-Q,18 MΩ.cm) made in the laboratory.

1.2 Preparation of Pt/C@NF and IrO_2@NF

A total of 7.13 mg Pt/C powder was dispersed in 100 μL deionized water, 80 μL of ethanol and 10 μL of 5 wt% Nafion solution through ultrasound for 30 min to form a uniform suspension. All of powder ink was loaded onto as-cleaned Ni foam (1×1 cm^2), followed with the dry in air at room temperature. The noble metal IrO_2@NF electrodes were prepared by the same method.

1.3 Material characterization

The surface morphology and internal structure of the samples were characterized by ultra-high resolution field emission scanning electron microscopy (SEM, JEOL JSM-7610F) and transmission electron microscopy (TEM, JEOL JEM-2100F), as well as the distribution of elements were characterized by energy dispersive X-ray spectroscopy (EDX). The phase composition of the samples was measured by X-ray diffractometer (XRD, Rigaku D/MAX 2200PC) with a Cu-Kα radiation source (λ = 0.15406 nm). The chemical valence and electronic structure of the samples were studied by X-ray photoelectron spectroscopy (XPS, Thermo Scientific EscaLab 250Xi) using Al-Kα radiation source (1486.6 eV).

1.4 Electrochemical measurements

All the prepared electrodes were electrochemically tested in Shanghai Chenhua Electrochemical Workstation (CHI 660E), and the electrolyte was an alkaline solution of 1 M KOH (PH = 14). The performance of HER and OER was tested by standard three-electrode system: the

prepared catalyst was the working electrode, the carbon rod was the counter electrode, and Hg/HgO (0.098 V) was the reference electrode. The area of working electrode immersed in electrolyte is 1×1 cm^2. The cyclic voltammetry (CV) and linear sweep voltammetry (LSV) with a sweep rate of 5 mV/s were carried out in the voltage range of -1.6~-0.9 V (HER) and 0~1.0 V (OER), and the Tafel slope and overpotential (η) were obtained by fitting and calculation. The reference electrode potential was transformed according to the reversible hydrogen electrode (RHE) formula:$E_{RHE} = E_{Hg/HgO} + 0.098 + 0.059 * PH$ [1] to show the actual IR compensation of catalysis. Electrochemical impedance spectroscopy (EIS) was tested at AC amplitude of 5 mV and frequency range of 0.01 Hz~100 KHz. The electrochemically active surface area (ECSA) of the catalyst was evaluated by electric double-layer capacitance (C_{dl}) by conducting CV tests at scan rates of 20, 40, 60, 80, and 100 mV/s in the non-Faradaic potential range. In addition, the overall water splitting performance was tested by double electrode system, and $P_{[0.9 mM]}$-MoS_2/Ni_3S_2@NF as the anode and cathode of alkaline electrolytic cell. The stability of the catalyst was evaluated using chronoamperometry (i-t) and long-term CV measurement.

2. Supplementary Figures:

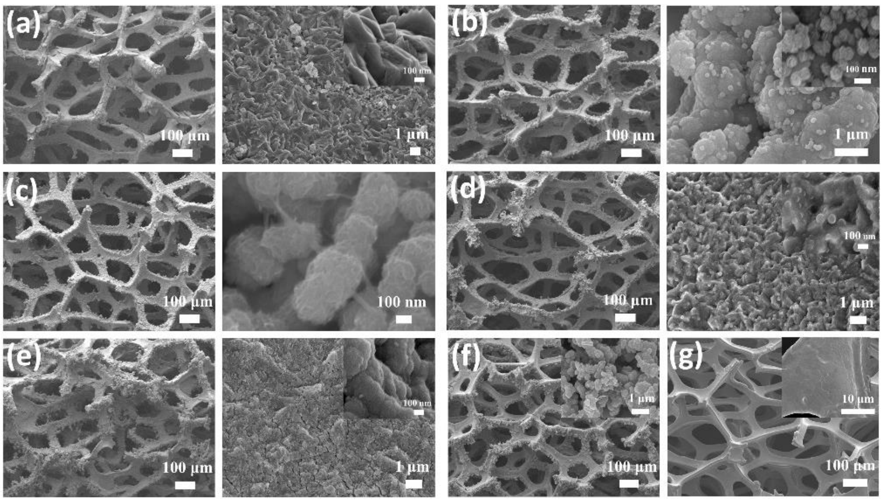

Figure S1. Low and high-resolution SEM images (a) $P_{[0.3 mM]}$-MoS_2/Ni_3S_2@NF, (b) $P_{[0.6 mM]}$-MoS_2/Ni_3S_2@NF, (c) $P_{[0.9 mM]}$-MoS_2/Ni_3S_2@NF, (d) $P_{[1.2 mM]}$-MoS_2/Ni_3S_2@NF, (e) $P_{[1.5 mM]}$-MoS_2/Ni_3S_2@NF, (f) MoS_2/Ni_3S_2@NF, and (g) carrier NF.

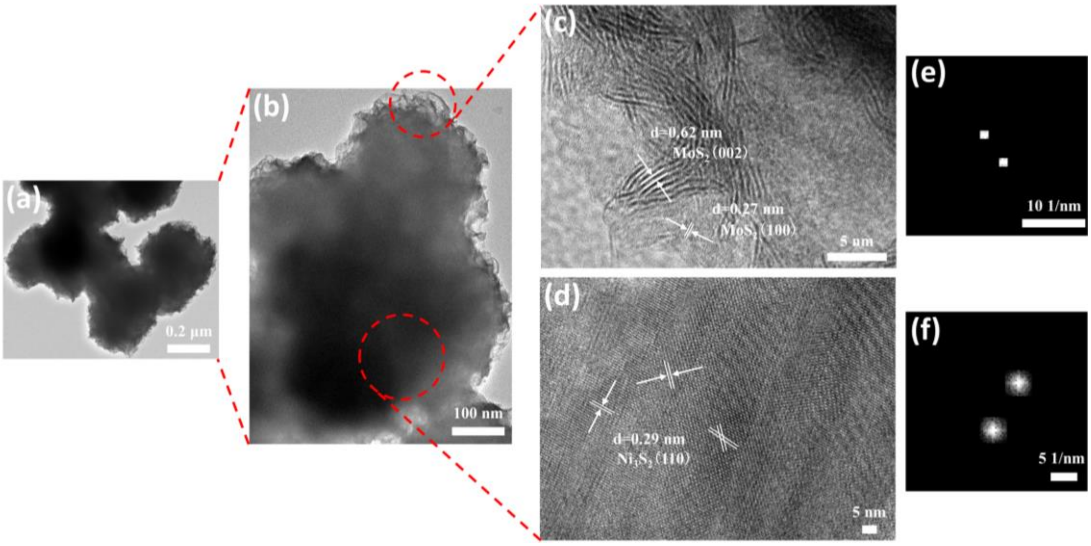

Figure S2. (a-b) TEM images, (c-d) HRTEM images, and corresponding (e-f) FFT patterns of $P_{[0.9\ mM]}$-MoS_2/Ni_3S_2@NF.

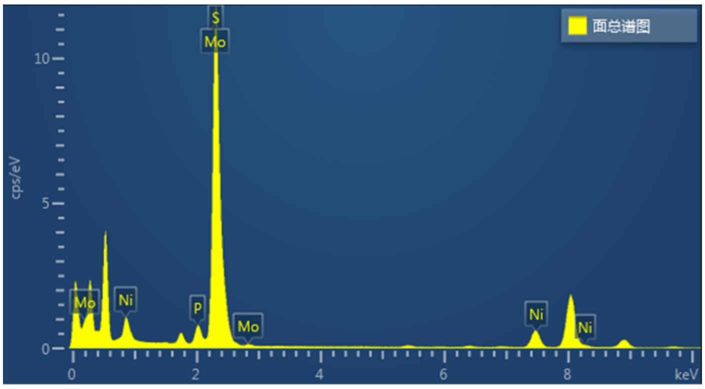

Element	wt%	wt% Sigma	Atomic percentage
P	2.06	0.06	4.08
S	24.81	0.34	47.40
Ni	4.48	0.07	4.67
Mo	68.66	0.37	43.85
Total	100.00		100.00

Figure S3. The EDX spectrum of $P_{[0.9\ mM]}$-MoS_2/Ni_3S_2@NF and the corresponding atomic (Ni, Mo, S, P) contents.

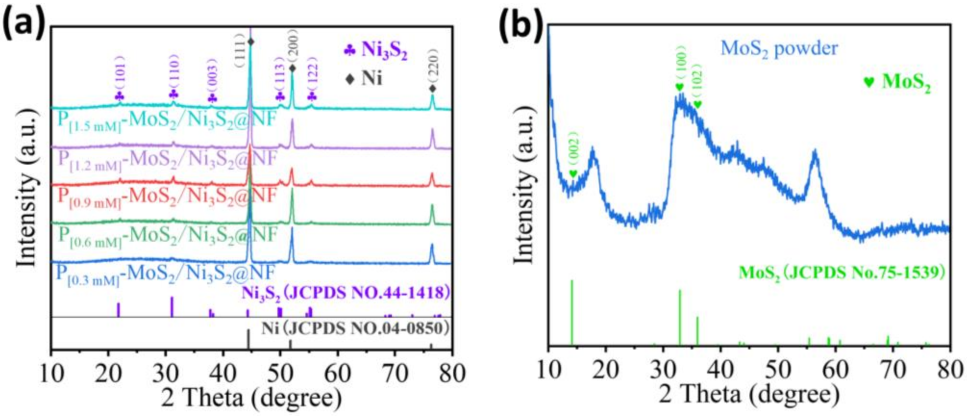

Figure S4. The XRD patterns of (a) catalyst electrodes doped with different P concentrations and (b) MoS_2 powders.

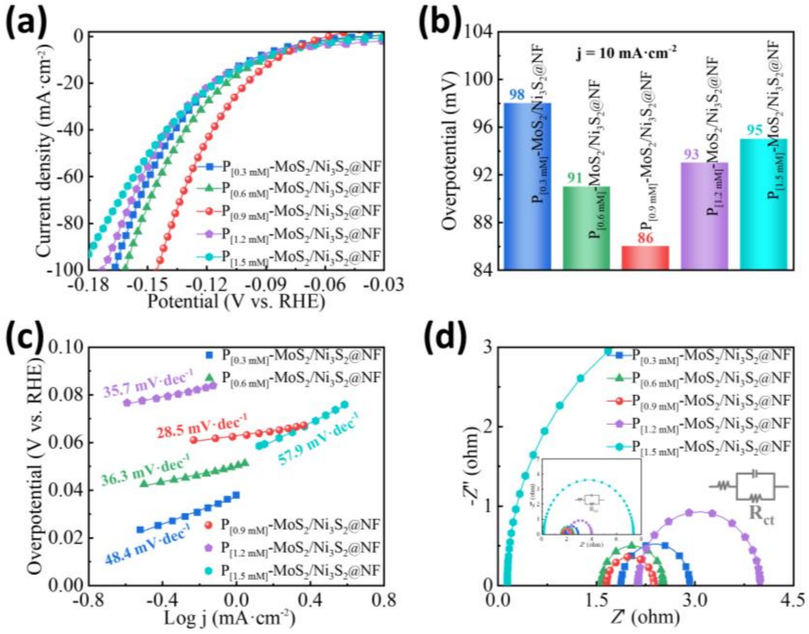

Figure S5. Comparison of HER performance of catalyst electrodes doped with different P concentrations (a) LSV curve, (b) overpotential histogram at a current density of 10 $mA\cdot cm^{-2}$, (c) Tafel slope, and (d) Nyquist curve.

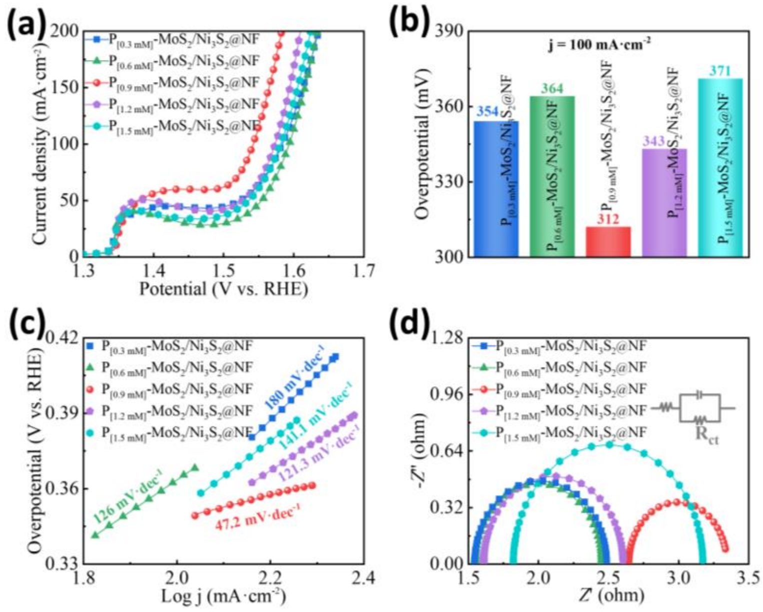

Figure S6. Comparison of OER performance of catalyst electrodes doped with different P concentrations (a) LSV curve, (b) overpotential histogram at a current density of 100 mA·cm^{-2}, (c) Tafel slope, and (d) Nyquist curve.

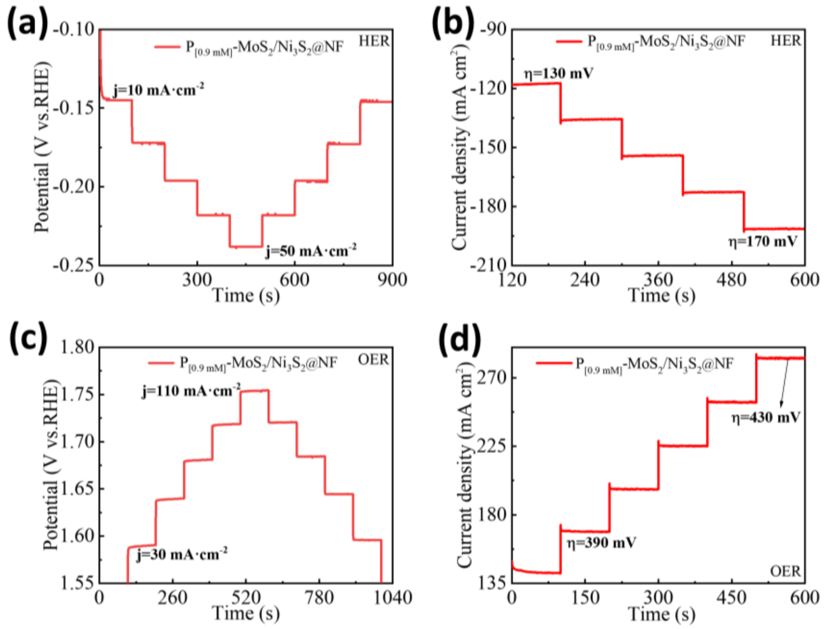

Figure S7. Multi-current and multi-potential steps for (a-b) HER, (c-d) OER.

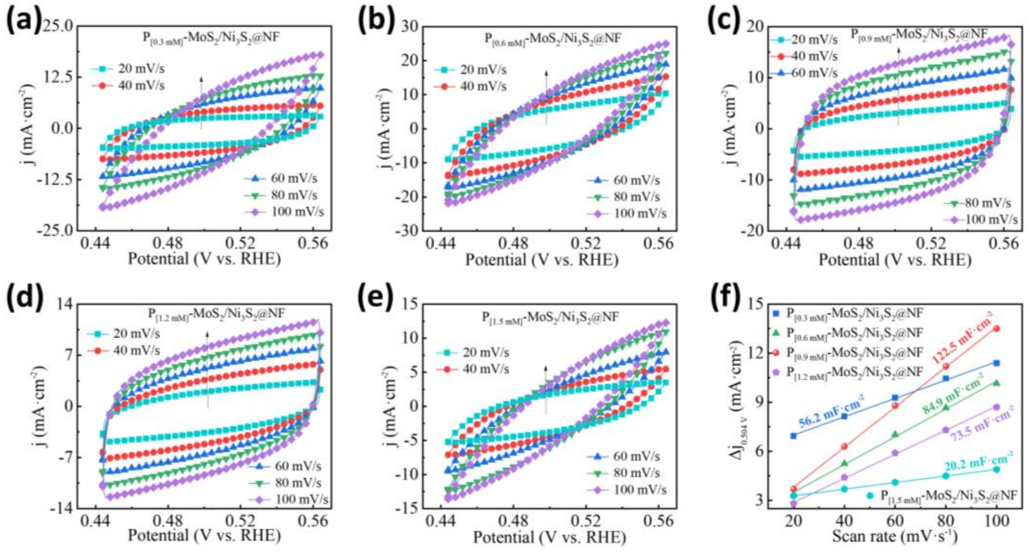

Figure S8. Comparison of electrocatalysts with different P doping concentrations, ECSA curves of (a) $P_{[0.3\ mM]}$-MoS_2/Ni_3S_2@NF, (b) $P_{[0.6\ mM]}$-MoS_2/Ni_3S_2@NF, (c) $P_{[0.9\ mM]}$-MoS_2/Ni_3S_2@NF, (d) $P_{[1.2\ mM]}$-MoS_2/Ni_3S_2@NF, (e) $P_{[1.5\ mM]}$-MoS_2/Ni_3S_2@NF, and (f) C_{dl} curves.

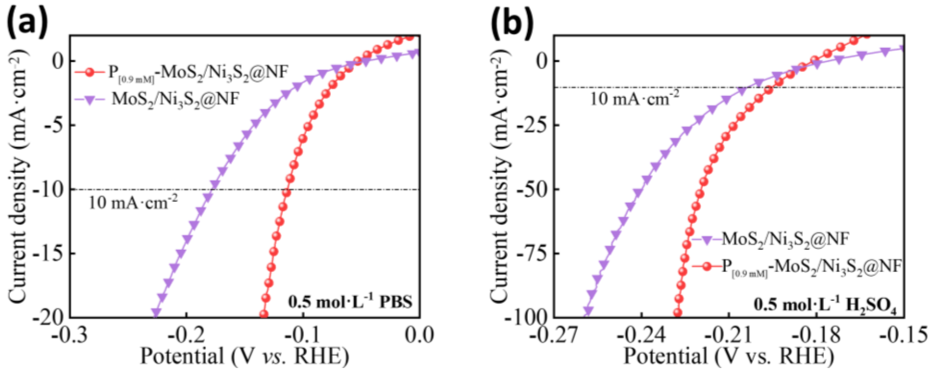

Figure S9. The electrocatalytic HER performance LSV curves of $P_{[0.9\ mM]}$-MoS_2/Ni_3S_2@NF in (a) neutral (0.5 M PBS, pH=7) and (b) acidic (0.5 M H_2SO_4, pH =0) electrolytes.

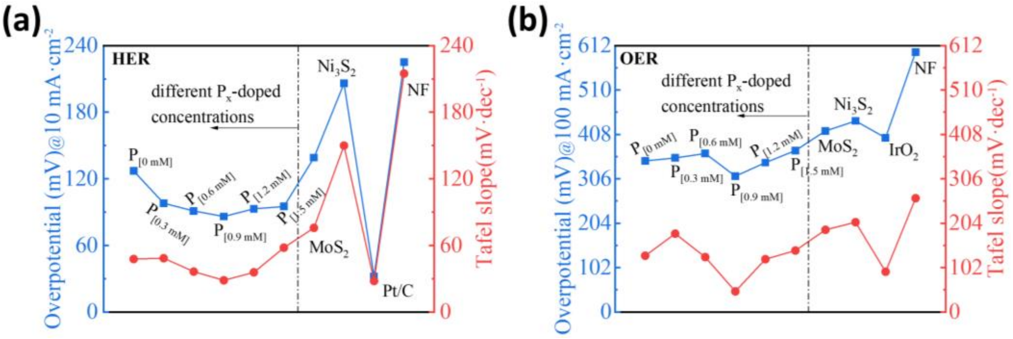

Figure S10. Comparison of overpotentials and Tafel slopes of as-prepared catalysts doped with different P concentrations and single-component (a) HER, (b) OER.

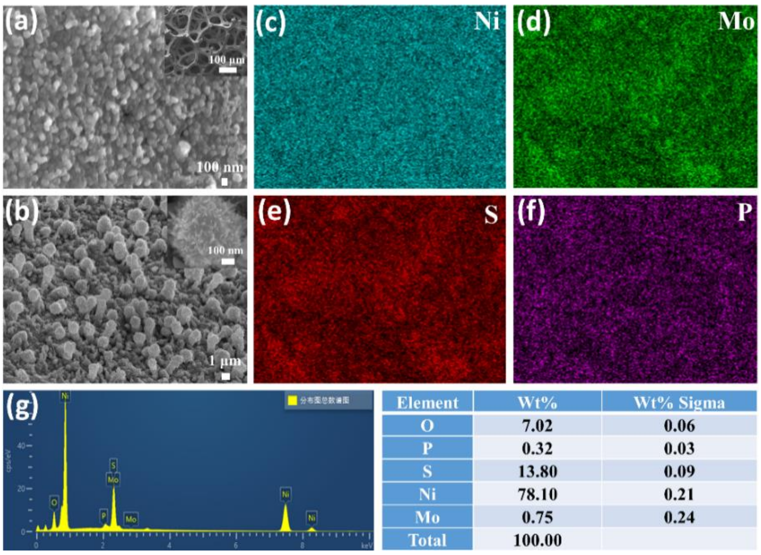

Element	Wt%	Wt% Sigma
O	7.02	0.06
P	0.32	0.03
S	13.80	0.09
Ni	78.10	0.21
Mo	0.75	0.24
Total	100.00	

Figure S11. After electrochemical testing of $P_{[0.9\ mM]}$-MoS_2/Ni_3S_2@NF (a-b) SEM images, (c-g) EDX mapping spectrum of corresponding elements (Ni, Mo, S, P).

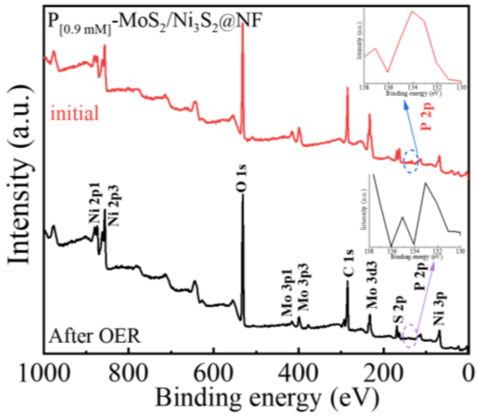

Figure S12. XPS full spectrum comparison before and after electrochemical OER testing of $P_{[0.9\ mM]}$-MoS_2/Ni_3S_2@NF.

3. Supplementary Tables:

Supplementary Table S1 Comparison of the electrocatalytic HER and OER performance of $P_{[0.9\ mM]}$-MoS_2/Ni_3S_2@NF and recently reported catalysts in alkaline electrolyte.

Electrocatalysts	HER		OER		Ref.
	η_{10}(mV)	Tafel slope (mV·dec^{-1})	η_{100}(mV)	Tafel slope (mV·dec^{-1})	
**$P_{[0.9\ mM]}$-MoS_2/Ni_3S_2@NF**	86	28.5	312	47.2	This work
**MOF-V-Ni_3S_2/NF**	118.1	113.2	268 / 10 mA·cm^{-2}	99	[1]
**MoS_2/Ni_3S_2 heterostructures**	110	83.1	218 / 10 mA·cm^{-2}	88	[2]
**Ni_3N-VN/NF Ni_2P-VP_2/NF**	64	37	398	49	[3]
**Ni_3S_2-300/Ni Foil**	135	75.7	319 / 20 mA·cm^{-2}	101.2	[4]
**MoS_2-Ni_3S_2 HNRs/NF**	98	61	249 / 10 mA·cm^{-2}	57	[5]
**Porous-MoS_2/Ni_3S_2/NF**	99	71	240 / 50 mA·cm^{-2}	46	[6]
**V-Ni_3S_2 nanowire**	68	112	--	--	[7]
**Fe-MoS_2/Ni_3S_2/NF-2**	130.6	112.7	320	59.5	[8]
**Fe, C- MoS_2/Ni_3S_2-450**	188	95	273 / 10 mA·cm^{-2}	66	[9]
**Co-N-Ni_3S_2/NF**	215	117.2	329	131.7	[10]
**N-MoS_x-Ni_3S_2-4@NF**	51	47	--	--	[11]
**Ni_3S_2@Ni nanorods**	82	73.8	339 / 20 mA·cm^{-2}	80.1	[12]
**Co_3S_4@MoS_2/Ni_3S_2**	136	72	270 / 50 mA·cm^{-2}	69	[13]
**NF/T(Ni_3S_2/MnS-O)**	116	41	228 / 10 mA·cm^{-2}	46	[14]
**CoS_2/MoS_2@CC**	71	62.8	340	57.5	[15]
**As-anodic CoS_x/Co**	102	92	362 / 50 mA·cm^{-2}	75.8	[16]

Supplementary Table S2 Comparison of R_{ct} and C_{dl} of prepared catalysts with different P concentrations and single-component catalysts

Electrocatalysts	HER-R_{ct}(ohm)	OER-R_{ct}(ohm)	C_{dl}($mF\cdot cm^{-2}$)
MoS_2/Ni_3S_2@NF	3.22	1.18	68.7
$P_{[0.3\ mM]}$-MoS_2/Ni_3S_2@NF	1.05	0.94	56.2
$P_{[0.6\ mM]}$-MoS_2/Ni_3S_2@NF	0.93	0.9	84.9
$P_{[0.9\ mM]}$-MoS_2/Ni_3S_2@NF	0.73	0.69	122.5
$P_{[1.2\ mM]}$-MoS_2/Ni_3S_2@NF	1.86	0.99	73.5
$P_{[1.5\ mM]}$-MoS_2/Ni_3S_2@NF	7.22	1.35	20.2
MoS_2@NF	9.37	1.69	27.9
Ni_3S_2@NF	13.59	1.87	15
Pt/C@NF<br>IrO_2@NF	0.24	1.35	--
NF	21.52	4.39	6.5

4. Notes and references

[1] Dong WX, Zhou HB, Mao BD, Zhang ZY, Liu YS, Liu YH, Li FH, Zhang DQ, Zhang DX, Shi WD. Efficient MOF-derived V-Ni_3S_2 nanosheet arrays for electrocatalytic overall water splitting in alkali. Int J Hydrogen Energy, 2021; 46: 10773-10782.

[2] Zhang J, Wang T, Pohl D, Rellinghaus B, Dong RH, Liu SH, Zhuang XD, Feng XL. Interface engineering of MoS_2/Ni_3S_2 heterostructures for highly enhanced electrochemical overall‐water‐splitting activity. Angew Chem 2016; 128: 6814-6819.

[3] Yan HJ, Xie Y, Wu AP, Cai ZC, Wang L, Tian CG, Zhang XM, Fu HG. Anion‐modulated HER and OER activities of 3D Ni-V-based interstitial compound heterojunctions for high‐efficiency and stable overall water splitting. Adv Mater 2019; 31: 1901174.

[4] Dong J, Zhang FQ, Yang Y, Zhang YB, He HL, Huang XF, Fan XJ, Zhang XM. (003)-Facet-

exposed Ni_3S_2 nanoporous thin films on nickel foil for efficient water splitting. Appl Catal B-Environ 2019; 243: 693-702.

[5] Yang YQ, Zhang K, Lin HL, Li X, Chan HC, Yang LC, Gao QS. MoS_2-Ni_3S_2 heteronanorods as efficient and stable bifunctional electrocatalysts for overall water splitting. ACS Catal 2017; 7: 2357-2366.

[6] Li F, Zhang DF, Xu RC, Fu WF, Lv XJ. Superhydrophilic heteroporous MoS_2/Ni_3S_2 for highly efficient electrocatalytic overall water splitting. ACS Appl Energy Mater 2018; 1: 3929-3936.

[7] Qu YJ, Yang MY, Chai JW, Tang Z, Shao MM, Kwok CT, Yang M, Wang ZY, Chua D, Wang SJ, Lu ZG, Pan H. Facile synthesis of vanadium-doped Ni_3S_2 nanowire arrays as active electrocatalyst for hydrogen evolution reaction. ACS Appl Mater Interfaces 2017; 9: 5959-5967.

[8] Xue JY, Li FL, Zhao ZY, Li C, Ni CY, Gu HW, Braunstein P, Huang XQ, Lang JP. A hierarchically-assembled Fe-MoS_2/Ni_3S_2/nickel foam electrocatalyst for efficient water splitting. Dalton Trans 2019; 48: 12186-12192.

[9] Lv XF, Liu GS, Liu S, Chen WT, Cao DH, Song TZ, Wang NN, Zhu YQ. Three-Dimensional Flower-like Fe, C-Doped-MoS_2/Ni_3S_2 Heterostructures Spheres for Accelerating Electrocatalytic Oxygen and Hydrogen Evolution. Crystals 2021; 11: 340.

[10] Du XQ, Ma GY, Zhang XS. Cobalt and nitrogen Co-doped Ni_3S_2 nanoflowers on nickel foam as high-efficiency electrocatalysts for overall water splitting in alkaline media. Dalton Trans 2021; 50: 8955-8962.

[11] Ma X, Chen WR, Li Q, Xue LJ, Peng C. Nitrogen-Doped Hierarchical Heterostructured Aerophobic MoS_x/Ni_3S_2 Nanowires by One‐pot Synthesis: System Engineering and Synergistic Effect in Electrocatalysis of Hydrogen Evolution Reaction. Energy Environ Mater 2021; 4: 658-663.

[12] Wu BX, Qian H, Nie ZW, Luo ZP, Wu ZX, Liu P, He H, Wu JH, Chen SG, Zhang FF. Ni_3S_2 nanorods growing directly on Ni foam for all-solid-state asymmetric supercapacitor and efficient overall water splitting. J Energy Chem 2020; 46: 178-186.

[13] Muthurasu A, Ojha GP, Lee M, Kim HY. Zeolitic imidazolate framework derived Co_3S_4 hybridized MoS_2-Ni_3S_2 heterointerface for electrochemical overall water splitting reactions. Electrochim Acta 2020; 334: 135537.

[14] Zhang Y, Fu JL, Zhao H, Jiang RJ, Tian F, Zhang RJ. Tremella-like Ni_3S_2/MnS with ultrathin nanosheets and abundant oxygen vacancies directly used for high speed overall water splitting. Appl Catal B 2019; 257: 117899.

[15] Zhou GY, Wu XM, Zhao MM, Pang H, Xu L, Yang J, Tang YW. Interfacial engineering ‐ triggered bifunctionality of CoS_2/MoS_2 nanocubes/nanosheet arrays for high ‐ efficiency overall water splitting. ChemSusChem 2021; 14: 699-708.

[16] Bian HD, Chen TY, Chen ZX, Liu JH, Li ZB, Du P, Zhou BB, Zeng XR, Tang JN, Liu C. One-step synthesis of mesoporous Cobalt sulfides (CoS_x) on the metal substrate as an efficient bifunctional electrode for overall water splitting. Electrochim Acta 2021; 389: 138786.
